# Disturbed brain ether lipid metabolism and histology in Sjögren‐Larsson syndrome

**DOI:** 10.1002/jimd.12275

**Published:** 2020-07-09

**Authors:** Pippa Staps, William B. Rizzo, Frédéric M. Vaz, Marianna Bugiani, Martin Giera, Bram Heijs, Antoine H. C. van Kampen, Mia L. Pras‐Raves, Marjolein Breur, Annemieke Groen, Sacha Ferdinandusse, Marinette van der Graaf, Gert Van Goethem, Martin Lammens, Ron A. Wevers, Michèl A. A. P. Willemsen

**Affiliations:** ^1^ Department of Pediatric Neurology, Radboud university medical center, Amalia Children's Hospital, Donders Institute for Brain Cognition and Behaviour Nijmegen Netherlands; ^2^ Department of Pediatrics, Child Health Research Institute University of Nebraska Medical Center Omaha Nebraska USA; ^3^ Laboratory Genetic Metabolic Diseases, Core Facility Metabolomics, Amsterdam UMC, University of Amsterdam Amsterdam Gastroenterology & Metabolism Amsterdam Netherlands; ^4^ Department of Pathology VU University Medical Center Amsterdam Netherlands; ^5^ Center for Proteomics & Metabolomics Leiden University Medical Center Leiden Netherlands; ^6^ Bioinformatics Laboratory, Department of Clinical Epidemiology, Biostatistics and Bioinformatics, Amsterdam Public Health research institute, Amsterdam UMC University of Amsterdam Netherlands; ^7^ Biosystems Data Analysis, Swammerdam Institute for Life Sciences University of Amsterdam Netherlands; ^8^ Department of Radiology and Nuclear Medicine Radboud University Medical Center Nijmegen Netherlands; ^9^ Department of Pediatrics, Radboud University Medical Center Amalia Children's Hospital Nijmegen Netherlands; ^10^ Het GielsBos, Gierle, Belgium and Department of Neurology University Hospital of Antwerp (UZA) Antwerp Belgium; ^11^ Department of Pathology Antwerp University Hospital, Edegem, and Laboratory of Neuropathology, Born‐Bunge Institute University of Antwerp Antwerp Belgium; ^12^ Department of Laboratory Medicine, Translational Metabolic Laboratory Radboud University Medical Center Nijmegen Netherlands

**Keywords:** brain, ether lipids, fatty aldehyde dehydrogenase, lipidomics, mass spectrometry imaging, odd‐chain fatty alcohols, pathology, phospholipids, Sjögren‐Larsson syndrome

## Abstract

Sjögren‐Larsson syndrome (SLS) is a rare neurometabolic syndrome caused by deficient fatty aldehyde dehydrogenase. Patients exhibit intellectual disability, spastic paraplegia, and ichthyosis. The accumulation of fatty alcohols and fatty aldehydes has been demonstrated in plasma and skin but never in brain. Brain magnetic resonance imaging and spectroscopy studies, however, have shown an abundant lipid peak in the white matter of patients with SLS, suggesting lipid accumulation in the brain as well. Using histopathology, mass spectrometry imaging, and lipidomics, we studied the morphology and the lipidome of a postmortem brain of a 65‐year‐old female patient with genetically confirmed SLS and compared the results with a matched control brain. Histopathological analyses revealed structural white matter abnormalities with the presence of small lipid droplets, deficient myelin, and astrogliosis. Biochemically, severely disturbed lipid profiles were found in both white and gray matter of the SLS brain, with accumulation of fatty alcohols and ether lipids. Particularly, long‐chain unsaturated ether lipid species accumulated, most prominently in white matter. Also, there was a striking accumulation of odd‐chain fatty alcohols and odd‐chain ether(phospho)lipids. Our results suggest that the central nervous system involvement in SLS is caused by the accumulation of fatty alcohols leading to a disbalance between ether lipid and glycero(phospho)lipid metabolism resulting in a profoundly disrupted brain lipidome. Our data show that SLS is not a pure leukoencephalopathy, but also a gray matter disease. Additionally, the histopathological abnormalities suggest that astrocytes and microglia might play a pivotal role in the underlying disease mechanism, possibly contributing to the impairment of myelin maintenance.

**SYNOPSIS**

The brain disorder in Sjögren‐Larsson syndrome (SLS) is explained by a severely disrupted lipid profile in both white and gray matter with accumulation of fatty alcohols and ether lipids and histopathological abnormalities in astrocytes and microglia.

## 1 INTRODUCTION

Sjögren‐Larsson syndrome (SLS; OMIM #270200) is a neurometabolic disorder caused by fatty aldehyde dehydrogenase (FALDH) deficiency^1^ due to biallelic mutations in *ALDH3A2*.^2^ Patients suffer from intellectual disability, spastic diplegia, ichthyosis, and retinopathy.^3^, ^4^ The FALDH deficiency results in accumulation of fatty aldehydes and fatty alcohols in plasma and skin.^5^, ^6^ FALDH is involved in the degradation of leukotrienes, which leads to increased levels of leukotriene B4 in patients.^7^ Furthermore, FALDH plays a role in phytol metabolism, converting phytenal into phytenic acid, apparently without accumulation of phytol or its degradation products in SLS patients.^8^ Brain magnetic resonance (MR) imaging studies show a white matter disorder,^9^, ^10^ and proton MR spectroscopy reveals abnormal signals between 0.8 and 1.6 ppm,^10^ which reflect abnormal lipid accumulation.^9^ Only a few postmortem reports on SLS are available, but in none of these patients, the diagnosis was biochemically or genetically confirmed.^11^, ^12^, ^13^, ^14^ Consequently, the underlying disease mechanisms causing the brain disorder in SLS, including the most important lipids involved, are not yet known.

**FIGURE 1:**
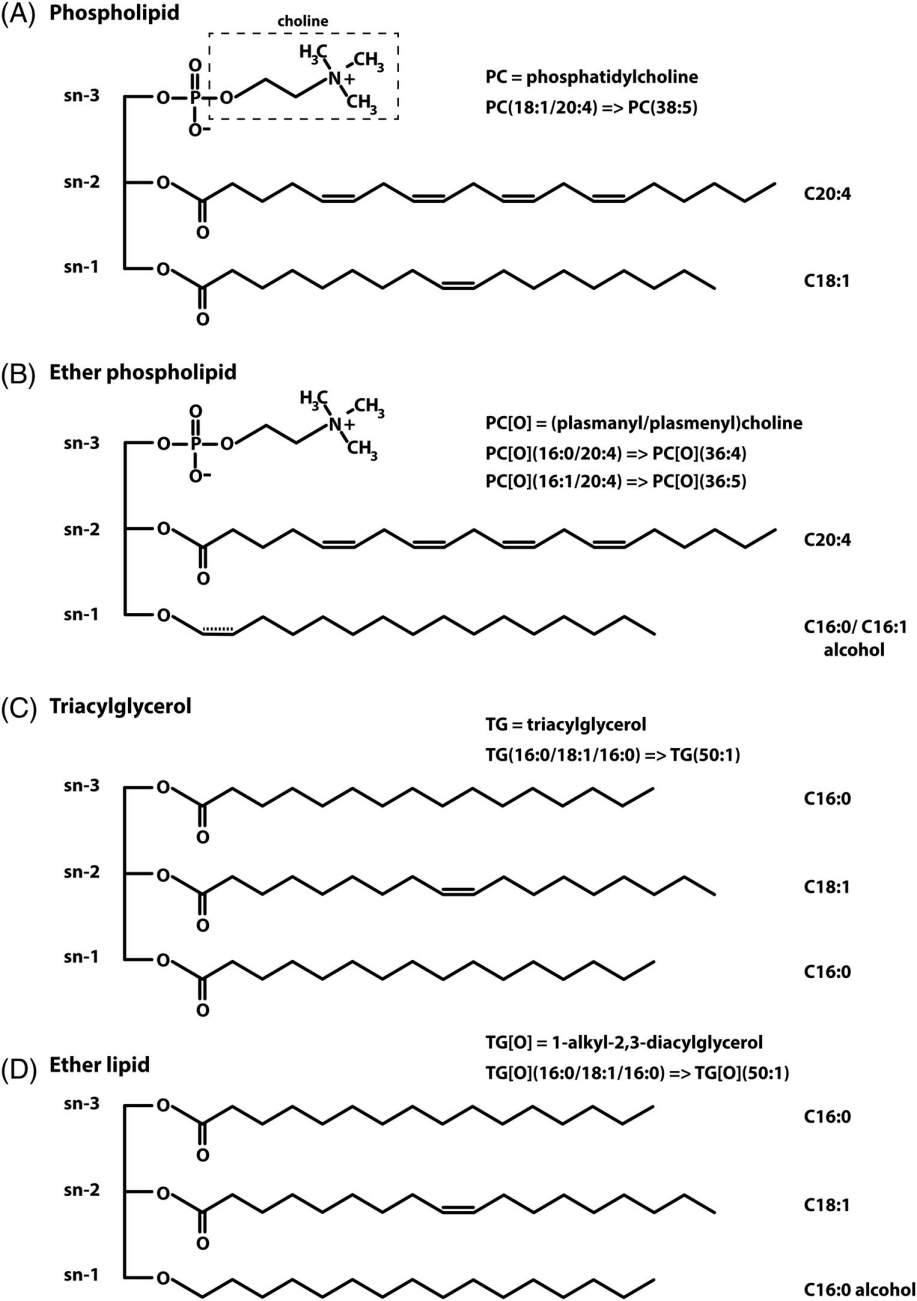
Relevant lipid structures and nomenclature. Lipid species are designated by their class followed by the sum of the carbon atoms in the fatty acid side chains and the total amount of double bonds. The main structure is shown for, A, phospholipids, in this case a phosphatidylcholine species (PC) with two fatty acids that have a total of 38 carbon atoms and 5 double bonds, for example, PC(38:5), B, ether phospholipids contain a fatty alcohol on the *sn*‐1 position of the glycerol backbone which results in an ether bond [O]. The species shown here has a choline headgroup and therefore is a plasm**a**nylcholine (PC[O]), and with this fatty acid/alcohol side chains is termed PC[O](36:4). A special class of ether phospholipids, plasmalogens, contain a vinyl‐ether on the *sn*‐1 position (extra double bond indicated in a dashed line). With the choline headgroup this class is called plasm**e**nylcholines, in this case PC[O](36:5). C, Triacylglycerols (TG) are the condensation product of three fatty acids and one glycerol molecule, here TG(50:1), whereas, D, 1‐alkyl‐2,3‐diacylglycerols, in analogy to ether phospholipids, have a ether‐linked fatty alcohol on the *sn*‐1 position (TG[O]), in this case TG[O](50:1)

Lipids comprise half of the human brain dry weight. They provide membrane structure and are required for membrane trafficking, signal transmission, and synaptogenesis.^15^ Cholesterol and phospholipids are the main membrane lipids. Phospholipids have a phosphate‐containing head‐group at the *sn‐*3 position of a glycerol backbone, and one or two fatty acyl‐groups linked via an ester bond. They are subdivided in subclasses based on the nature of the head group (ie, phosphatidylethanolamine contains an ethanolamine head‐group, whereas phosphatidylcholine contains a choline head group).^15^ Ether phospholipids, including plasmalogens, constitute a special phospholipid class, characterized by the presence of an ether or a vinyl ether at the *sn‐*1 position of the glycerol backbone instead of an ester (Figure 1).^16^ The most abundant lipid species in the human brain, apart from cholesterol, are phospholipids with phosphatidylcholine (PC), phosphatidylethanolamine (PE), and phosphatidylserine (PS) as the three most abundant classes.^17^

We studied the postmortem brain of a 65‐year‐old female patient with genetically confirmed SLS using targeted as well as untargeted lipidomic approaches in combination with morphological analyses. Our results show a severely disturbed lipid profile in the SLS brain with accumulation of various species of fatty alcohols, ether(phospho)lipids, and triacylglycerols in both white and gray matter, and confirm the presence of structural white matter abnormalities.

## 2 MATERIALS AND METHODS

Materials and methods are described in the Supporting information.

## 3 RESULTS

### 3.1 ResultsHistopathology of the SLS brain

**FIGURE 2:**
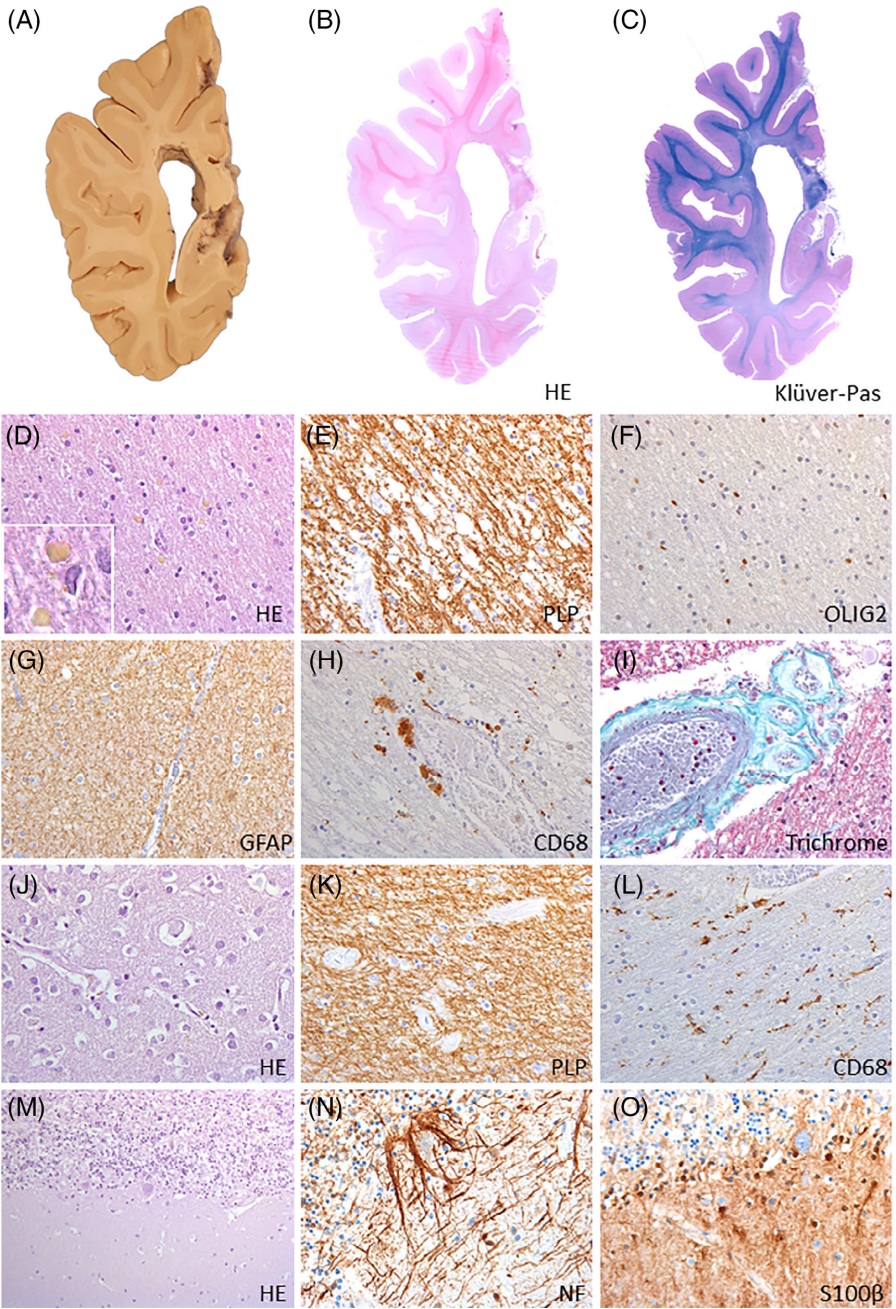
Neuropathology of SLS. A, Whole coronal slice of the right hemisphere at the level of the pulvinar shows no normal trophism of the cortex and of the white matter. B,C, Whole coronal mounts at the same level show that the periventricular and deep hemispheric white matter is pale (B, hematoxylin and eosin [H&E]; C, Kluver stain for myelin combined with periodic acid Shiff), whereas the U‐fibers are relatively better preserved. D, H&E stain of the occipital white matter confirms white matter pallor and reveals preservation of oligodendrocyte numbers and presence in the neuropil of yellowish lipid droplets (inset). E, Stain for the major myelin protein proteolipid protein (PLP) shows that white matter pallor is due to decreased amounts of myelin. F, stain for the pan‐oligodendrocytic marker Olig2 further confirms that oligodendrocytes are not depleted. G, Stain against the astrocyte marker glial fibrillary acidic protein (GFAP) reveals a diffuse isomorphic astrogliosis. H, stain against the microglia/macrophage marker CD68 shows that cells with a phagocytic morphology are clustered around blood vessels. I, the histochemical stain Trichrome shows blood vessel wall changes consistent with hyalinosis, including thickening of the walls and loss of cells in the tunica media. J, H&E stain of the occipital cortex shows normal organization with no loss of neurons. K, PLP stain of the descending tracts in the brainstem shows reduced myelin amounts. L, In the same areas, the CD68 stain reveals presence of normal numbers of microglia that do not show an ameboid activated aspect. M,N, H&E stain (M) and immunohistochemistry against high‐molecular weight neurofilaments (NF, N) reveals loss of Purkinje cells in the cerebellar cortex with presence of empty baskets. O, Stain against the Bergmann glia marker S100β shows that Purkinje cell dropout corresponds with proliferation of Bergmann glia, some of which are abnormally translocated to the molecular layer

The SLS brain (weight 1347 g) was macroscopically normal (Figure 2A‐C). Microscopically, lack of myelin with tissue rarefaction was seen in hemispheric deep white matter and capsulae (Figure 2D,E), whereas the U‐fibers and corpus callosum were better preserved without signs of demyelination. Oligodendrocyte numbers were not diminished and scattered axonal spheroids were found (Figure 2D‐F). A diffuse isomorphic astrocytic gliosis was present, and some microglia had a phagocytic morphology (Figure 2G,H). Around blood vessels clustering macrophages contained pigmented PAS‐positive iron‐negative material. These pathological changes were more evident in occipital white matter. In the white matter, smaller blood vessels had thickened walls with relative loss of smooth muscle cells in the tunica media and thickened tunica adventitia (Figure 2I). The cerebral cortex was only mildly gliotic, without neuronal dropout or microglia activation. Basal nuclei, thalamus, and hippocampus were unaffected (Figure 2J). The long white matter tracts in the brainstem and spinal cord also showed deficient myelin, however, without microglia or astrocytic activation (Figure 2K,L). The cerebellar white matter was better preserved compared to the cerebral areas; however, activated microglia and scattered macrophages were found in perivascular spaces. In cerebellar cortex, a mild dropout of Purkinje cells and granular neurons were seen, with some degree of mislocalization of the Bergmann glia nuclei to the molecular layer (Figure 2M‐O). No white matter pathology was found in the optic nerve, optic chiasm, and optic tract.

### 3.2 Free fatty alcohols and fatty aldehydes

**FIGURE 3:**
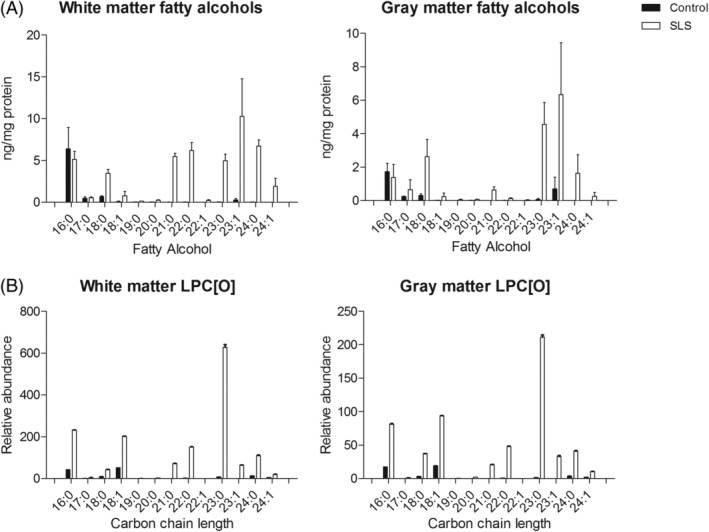
Fatty alcohols and lysophosphatidylcholine etherphospholipid abundancies in SLS and control brain tissue. A, Mean concentrations of fatty alcohols are shown in both white and gray matter of SLS and control brain tissues. Fatty alcohols were measured in four separate samplings of brain (mean, SEM). B, Mean abundancies of individual LPC[O]s as measured with untargeted lipidomics; brain samples were measured in triplicate using three pieces of the same brain samples that were processed independently. LPC[O], lysophosphatidylcholine etherphospholipid; SLS, Sjögren‐Larsson syndrome

The total free fatty alcohols as a group (C16‐24) were increased in SLS brain compared to control brain by 4.1‐fold in white matter and 3.8‐fold in gray matter. A similar fatty alcohol profile was observed for white and gray matter of the SLS brain but fatty alcohols accumulated in much higher concentrations in white matter than in gray matter (Figure 3A). The chain lengths of the accumulating alcohols ranged from C18 to C24 and comprised both even‐ and odd‐length chains. Notably, the long‐chain fatty alcohols C21 to C24, both saturated and mono‐unsaturated were highly elevated in SLS brain, more so in white matter than gray matter. In SLS white matter, this subgroup of fatty alcohols was increased by 17‐fold over control brain. C23‐fatty alcohols were the most abundant species in both white and gray matter, with fold changes of respectively 166 and 76. There was no parallel accumulation of free very long chain fatty acids. Surprisingly, although small amounts of free fatty aldehydes (C14:0, C16:0, C17:0, C18:0, and C18:1) were present in both brains, there was no accumulation of aldehydes in the SLS brain (data not shown).

### 3.3 Lipid analysis

**FIGURE 4:**
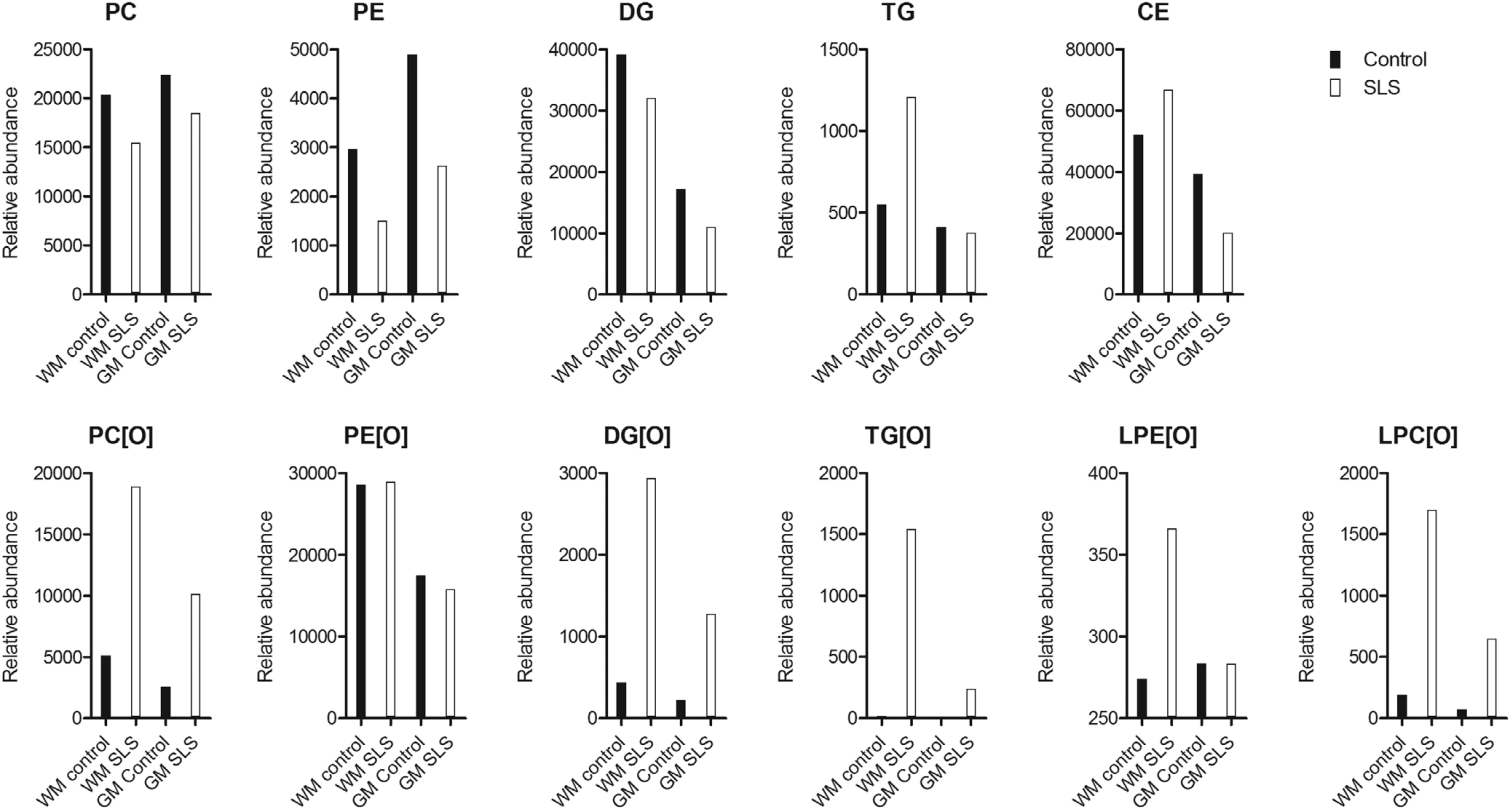
Abundancies of different lipid classes in SLS and control brain tissue. Totals of the quantitatively most important lipid species per major class for control and patient white and gray matter; the mean abundance is shown; Brain samples were measured in triplicate using three pieces of the same brain samples that were processed independently. CE, cholesteryl ester; DG, diacylglycerol; DG[O], 1‐alkyl‐2‐acylglycerol; PC, phosphatidylcholine; (L)PC[O], (lyso)phosphatidylcholine etherphospholipid; PE, phosphatidylethanolamine; (L)PE[O], (lyso)phosphatidylethanolamine etherphospholipid; SLS, Sjögren‐Larsson syndrome; TG, triacylglycerol; TG[O], 1‐alkyl‐2,3‐diacylglycerol

The lipidome of white and gray matter samples of SLS and control brain was using untargeted lipidomics. A total of almost 1700 individual lipid species from 33 different lipid classes could be evaluated by our lipidomics technique. The most abundant lipid classes in the control brain in descending order are cholesterol and its esters, DG, PE[O], PC[O], DG[O], TG, CL, BMP, LPE[O], and LPC[O] (Figures 3B and 4). The profile was similar for white and gray matter. Most individual lipid species were found in remarkably similar concentrations in SLS and control brain, suggesting that the quality of both brain samples and the analysis was comparable. The following major classes, including subspecies, did not change significantly when comparing SLS and control brain: sphingolipids; ceramides, ceramide‐1‐phosphates, hexosylceramides (HexCer), lactosylceramides, sulfatides, hydroxysulfatides, sphingosines, sphingosine‐1‐phosphates and sphingomyelins, and phospholipids; (lyso)phosphatidic acids, (lyso)phosphatidylglycerols, phosphatidylinositols, phosphatidylserines, and mono‐ and dilysocardiolipins.

Several lipid classes accumulated in SLS brain, both in white and gray matter (Figure 4). The majority of the accumulating lipid classes were ether lipids (DG[O], TG[O]) and ether phospholipids (mainly PC[O], LPC[O]), whereas the corresponding non‐ether lipid levels were not elevated (DG, PC, LPC), or even reduced, with the exception of TG levels in white matter which were higher in SLS. Surprisingly, the ethanolamine ether phospholipids, PE[O] and LPE[O] as a group, were unchanged in both SLS white and gray matter. To investigate if the changes in the ether phospholipid levels impacted the plasmanyl/plasmenyl distribution (ie, ether phospholipid vs plasmalogen), we compared the levels of representative and well detected PE[O] and PC[O] species with‐ and without hydrochloric acid treatment. This treatment hydrolyses the alkenyl‐bond (ie, plasmenyl) but not the alkyl (ie, plasmanyl) species on the sn‐1 position. The plasmanyl/plasmenyl ratio was consistently unchanged for both PC[O] and PE[O] when comparing SLS and control brain for both white and gray matter (Supporting information). Other non‐ether phospholipid classes including PS, LPE, and PE also showed a trend toward lower levels in SLS white matter, respectively, 77%, 59%, and 55% of the control tissue abundancy. In gray matter, CE and DG showed a trend toward lower levels in SLS, with, respectively, 51% and 64% abundancy of the control tissue. The concentration of accumulating ether lipids was highest in SLS white matter. The highest fold change (FC_SLS/Control_) was 93 for TG[O]. Other grossly elevated lipid classes were LPC[O] (FC_SLS/Control_ = 9.0), DG[O] (FC_SLS/Control_ = 6.8), and PC[O] (FC_SLS/Control_ = 3.7). The lipid classes that contributed most to the abnormal lipid accumulation in the SLS white matter on a molar basis are in descending order CE, PC[O], DG[O], LPC[O], TG[O], and TG which was similar to what was observed in SLS gray matter although the concentration of the accumulating lipid classes was less pronounced in gray matter (Figure 4).

#### 3.3.1 Fatty alcohols in lysoforms of ether phospholipids

Fatty alcohols are used for the synthesis of the 1‐*O*‐alkyl chain in ether (phospho)lipids. Figure 3B shows the abundance of LPC[O] species in control‐ and SLS brain. In these lipids, long‐chain fatty alcohol species predominated both in white and gray matter. A similar pattern of carbon chain lengths occurred in fatty alcohols and LPC[O]'s and to a lesser extent in LPE[O]'s. In LPC[O], the chain length of the accumulating species varied between C16‐C24 and, somewhat unexpectedly, the most abundant LPC[O] was LPC[O] 23:0. LPC[O] 23:1 accumulated to a lesser extent than in its fatty alcohol counterpart. Collectively, these data indicate that the pattern of accumulating fatty alcohol species is very similar to the distribution of LPC[O] species and that very long‐chain, odd‐chain fatty alcohols are the main accumulating fatty alcohols.

#### 3.3.2 Individual lipid subspecies

To evaluate which specific lipid species predominantly accumulate in SLS brain, we considered individual lipids with a fold change >3 in SLS compared to control and a difference in relative abundance >10. A total of 175 individual lipids from 11 lipid classes fulfilled these criteria in white matter, compared to 104 individual lipids from 9 lipid classes in gray matter (Supporting information). For most lipid classes, the accumulating lipid subspecies overlapped between white and gray matter (LPC[O], PC[O], DG[O], TG[O]). Of all TG[O] species detected in SLS white matter, 69% had more than 54 carbons in the fatty acid side chains (more than three C18). In SLS white matter, the individual lipid species with the highest fold‐change compared to control were CE(24:4) (FC_SLS/Control_ = 10.9), PC[O](32:0) (FC_SLS/Control_ = 7.1), LPC[O](23:0) (FC_SLS/Control_ = 84.2), and TG[O](52:2) (FC_SLS/Control_ = 127). In gray matter, these were CE(24:5) (FC_SLS/Control_ = 3.8), PC[O](39:0) (FC_SLS/Control_ = 250.5), LPC[O](23:0) (FC_SLS/Control_ = 118.2), and DG[O](39:1) (FC_SLS/Control_ = 13.8). In almost all cases, the abundance of accumulating ether lipid species in SLS was clearly higher in white than in gray matter, with exception of PE[O]. Remarkably, the abundance of selected odd‐chain PE[O] species, however, was clearly higher in SLS gray matter than in white matter, namely PE[O](45:8), PE[O] (45:7), and PE[O](45:6), accumulated in gray matter with fold changes between 14 and 360 but not in white matter (Supporting information). When comparing the sum of all PE[O] species between SLS and control, no large increase was seen as opposed to other ether lipid species. In gray matter, 26 PE[O] species fulfilled the criteria of a fold change >3 and an abundance >10 compared with control brain. Almost all of these PE[O] species (n = 25 of 26) contained (very) long fatty acids/alcohols with a total of carbon atoms ranging from 39 to 47 and were polyunsaturated (17 out of 24 had 5 or more double bonds). Although the sum of the PE[O] species was not increased in SLS white matter, individual long‐chain PE[O] species did accumulate when compared to control, even more than in gray matter (seven PE[O] species with chain length 46 and 47 and 5‐8 double bonds; Supporting information). The total CE level in control white matter was only moderately elevated in SLS brain, but this was not seen in gray matter (Figure 4). Remarkably, specific CE species with polyunsaturated long‐ and very‐long‐chain acyl‐groups were highly elevated in SLS white matter (20:2, 22:3, 24:5, 22:5, 24:4) and to a lesser extent also in gray matter (Supporting information).

### 3.4 Mass spectrometry imaging

**FIGURE 5:**
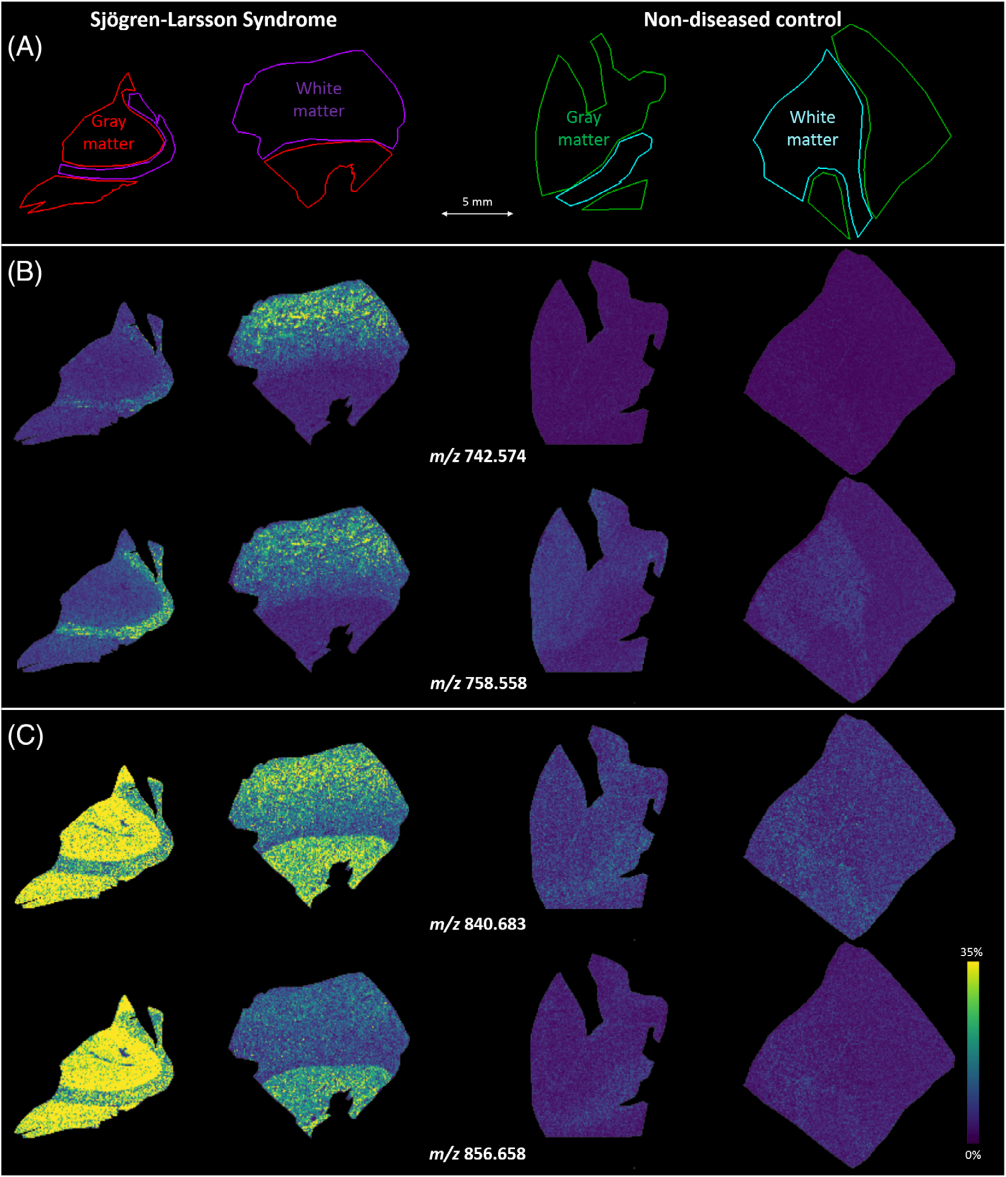
Mass spectrometry imaging‐based distributions of ether‐phospholipids in SLS and control brain tissue. A, Overview of the tissue morphology. Gray matter areas from the SLS brain (red) and control (green), as well as white matter areas from the SLS brain (purple) and control brain (cyan) were measured. B, In‐situ distributions of PC(O‐32:0) detected as both Na^+^‐adduct (*m/z* 742.574) and K^+^‐adduct (*m/z* 758.558). C, In‐situ distributions of PC(O‐39:0) detected as both Na^+^‐adduct (*m/z* 840.683) and K^+^‐adduct (*m/z* 856.658). PC[O], phosphatidylcholine etherphospholipid; SLS, Sjögren‐Larsson syndrome

During sectioning of brain tissue for mass spectrometry imaging, two blocks from each brain were prepared; one containing mostly gray matter, the other containing mostly white matter. As expected, large differences in the lipid content were visible between white and gray matter, both in the SLS and control tissues (Supporting information). A total of 1872 *m/z* features were detected from the overall mass spectrum (including both white and gray matter regions, from SLS and control tissues). We searched for the most discriminating peaks between the SLS and control tissues. The lipid features that distinguish the SLS and control profile could be tentatively identified as PE[O] and PC[O] species. The differentiating lipid species were low in abundance, but had a unique presence in the SLS patient brain. PC[O](32:0) and PC[O](39:0) clearly accumulated in the SLS brain (Figure 5). This is in line with the biochemical data from the untargeted lipidomics approach in the previous section and in Supporting information. The same holds true for the accumulation of individual PE[O] species.

## 4 DISCUSSION

Our study describes a comprehensive biochemical as well as histopathological evaluation of the postmortem brain of a patient with genetically confirmed SLS; the results contribute to our understanding of SLS, and illustrate the power of novel techniques to clarify the biochemical abnormalities of the brain in neurometabolic disorders.

The white matter of the SLS brain showed lack of myelin and perivascular macrophages containing pigmented lipoid material, with a posterior hemispheric predominance. The absence of numerous, scattered macrophages in the parenchyma argues against ongoing demyelination and rather suggests an impairment in the process of myelin maintenance. The white matter abnormalities and accumulating lipoid material correspond to the results of cerebral MR imaging and spectroscopy in living SLS patients.^10^ Interestingly, in the more affected occipital lobes, we also found astrocyte and microglia activation, suggesting that the disease mechanisms underlying the leukodystrophy are ongoing. Dysfunction of astrocytes and microglia could contribute to an impairment in myelin maintenance.^18^ The direct involvement of these cell types is in line with the findings in previous retinal studies in SLS, which suggested a similar role for Müller cells, the retinal counterpart of cerebral astrocytes.^19^

**FIGURE 6:**
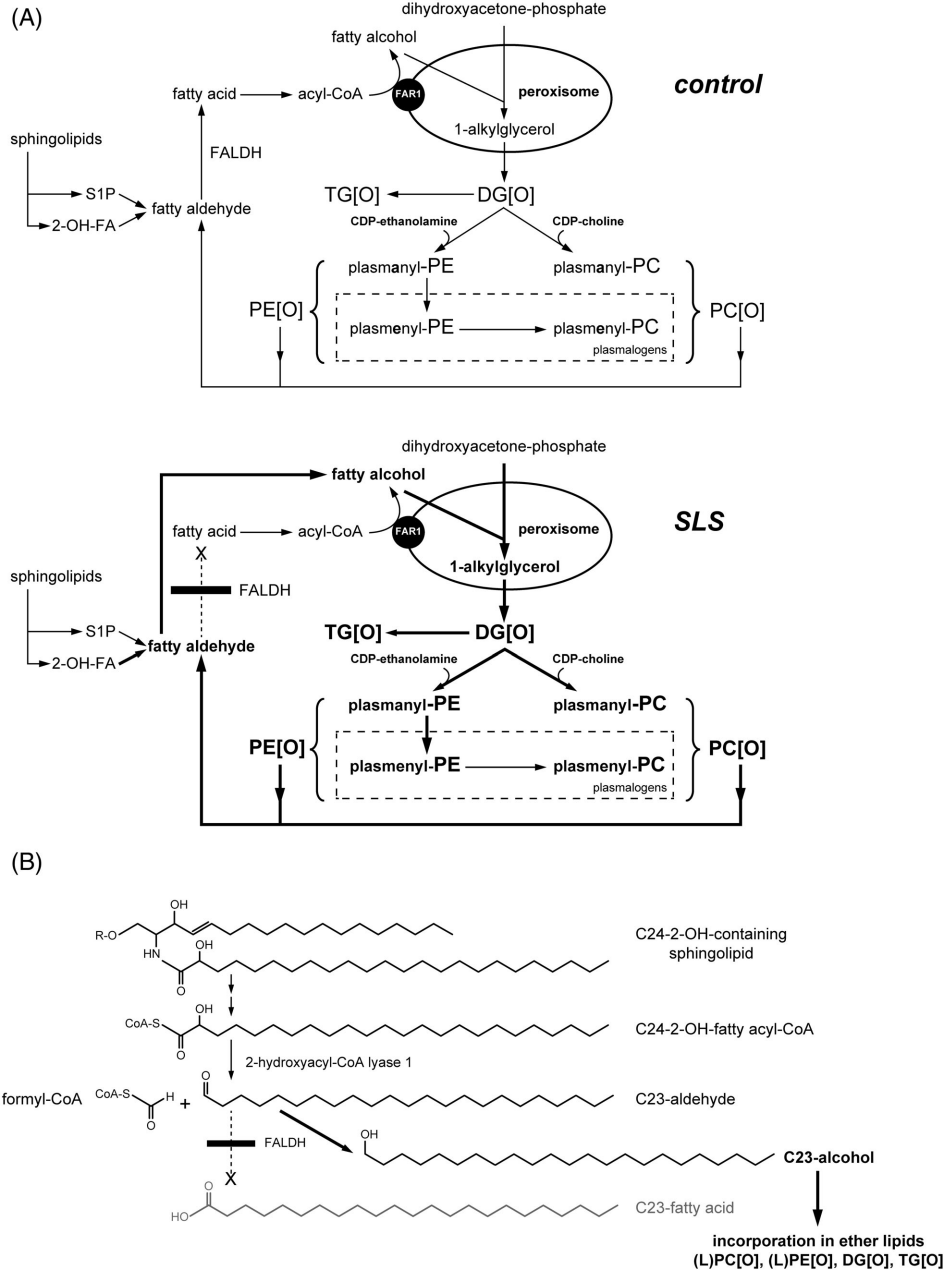
Proposed origin of accumulating ether lipids in SLS. A, Etherphospholipid metabolism in the control brain (upper panel), and the SLS brain (lower panel). Dihydroxyacetone phosphate and a fatty alcohol is converted to alkylglycerol in the peroxisome after which alkylglycerol is acylated to form 1‐alkyl‐2‐acylglycerol (DG[O]). DG[O] can be converted to PE[O] and PC[O] by condensation with CDP‐ethanolamine and CDP‐choline, forming plasmanyl‐PE and plasmanyl‐PC. Creation of a vinyl‐ether by desaturation, yields plasmenyl‐PE/PC. Collectively the plasmanyl‐ and plasmenyl forms are referred to as plasmalogens. DG[O] can also be acylated yielding 1‐alkyl‐2,3‐diacylglycerol (TG[O]). Catabolic aldehydes are shown which normally are converted to fatty acids by FALDH. In case of FALDH deficiency in SLS, accumulating fatty aldehydes are converted to fatty alcohols which are then incorporated in the ether‐(phospho)lipids DG[O], TG[O], PC[O], and PE[O]. B, Hypothetic metabolic pathway explaining the formation and accumulation of odd chain fatty alcohols and odd‐chain etherlipid and etherphospholipid species in the SLS brain. The accumulation of these odd chain lipid species is explained using the example of the conversion of a C24‐2‐hydroxy containing sphingolipid. After liberation of its fatty acyl chain and activation by CoA‐addition a C24‐2‐OH‐CoA results. It is subsequently cleaved by 2‐hydroxyacyl‐CoA lyase 1 yielding formyl‐CoA and a C23‐aldehyde. Due to the FALDH deficiency, this C23‐aldehyde is reduced to its corresponding alcohol and incorporated into etherlipids and etherphospholipids. DG[O], 1‐alkyl‐2‐acylglycerol; FALDH, fatty aldehyde dehydrogenase; FAR1, Fatty Acyl‐CoA Reductase 1; (L)PC[O], (lyso)phosphatidylcholine etherphospholipid; PE[O], phosphatidylethanolamine etherphospholipid; SLS, Sjögren‐Larsson syndrome; TG[O], 1‐alkyl‐2,3‐diacylglycerol

It is known that fatty alcohols accumulate in body fluids of SLS patients,^1^ but their accumulation in the central nervous system has never been shown. We found a striking accumulation of fatty alcohols in SLS brain with a predominance of C18‐C24 chain length species. Interestingly, although the C18:0 alcohol was elevated, the C16:0 alcohol was not. This long‐chain fatty alcohol pattern differs from that seen in SLS plasma, cultured fibroblasts and keratinocytes in which only C16‐C18 fatty alcohols accumulate.^5^, ^20^ The longer chain fatty alcohols (C21‐C24) therefore likely are unique to brain. Long‐chain fatty alcohols are synthesized from their corresponding fatty acids of similar chain length but can also be formed in the fatty alcohol cycle by reduction of aldehydes that originate from catabolism of metabolites, which will be discussed below.^21^, ^22^ The fatty acyl‐CoA‐reductase (FAR) enzyme in mouse and bovine, involved in the de novo synthesis of fatty alcohols, has highest activity with C15‐C18 acyl‐CoA substrates and very low activity with C20‐C22‐acyl‐CoA substrates.^23^, ^24^ Humans possess two distinct FAR enzymes (FAR1 and FAR2); it is unknown which of these two enzymes is responsible for synthesizing the longer C21‐C24 alcohols identified in SLS brain, and whether or not they are synthesized via this pathway at all.^25^ Nevertheless, the normal fatty acid composition of the SLS brain argues that the accumulation of the fatty alcohols is not due to an increase in their rate of synthesis via either FAR1 or FAR2. Another potential source of fatty alcohols is the catabolism of different metabolites that lead to the production of fatty aldehydes via the fatty alcohol cycle. Fatty aldehydes arise from the degradation of plasmalogens, sphingosine‐1‐phosphate, branched‐chain fatty acids and 2‐hydroxy‐fatty acids (Figure 6A).^22^, ^26^ Aldehydes that are usually oxidized to fatty acids by FALDH would accumulate and be reduced to fatty alcohols, a phenomenon that was previously observed in SLS.^1^ In this respect, the predominant accumulation of the odd‐chain C23‐alcohol was intriguing and unexpected. We speculate that this originates from the breakdown of 2‐hydroxy‐fatty acids derived from sphingolipids carrying these 2‐hydroxy‐fatty acids, which are very abundant in brain, especially in white matter.^17^, ^27^ When liberated, 2‐hydroxy‐fatty acids are activated to their corresponding CoA‐ester and the enzyme 2‐hydroxyacyl‐CoA lyase 1 cleaves this molecule yielding formyl‐CoA and an n‐1 aldehyde.^28^ In brain, the most abundant 2‐hydroxy‐fatty acid is C24,^27^ which results in the formation of C23‐aldehyde when broken‐down via this route (Figure 6B). As FALDH has the ability to metabolize aliphatic aldehydes ranging from 6‐ to 24‐carbons long, with substrate specificity toward long‐chain fatty aldehydes we believe that C23‐aldehyde could also be a substrate for FALDH and that this thus accumulates in SLS.^29^, ^30^ FALDH functions as part of a complex that, together with fatty alcohol dehydrogenase, sequentially converts fatty alcohols to fatty aldehydes and fatty acids.^1^, ^31^ Since fatty alcohols are metabolic precursors for synthesis of ether lipids, it is likely that the accumulation of LPC[O] and TG[O] in SLS brain is a direct consequence of increased amounts of fatty alcohol substrates (Figure 6B) and that this distribution reflects the accumulating alcohols and those resulting from reduction of catabolically formed aldehydes. There was no increase in plasmalogens in SLS brain based on the dimethyl acetal peaks seen on fatty acid analysis. Although the C18:0 alcohol accumulated in the SLS brain and is a substrate for plasmalogen synthesis, it does not seem to drive the synthesis of plasmalogens by itself. This was supported by the lipidomics experiment which showed comparable levels of plasmenyl‐species in SLS and control brain (Supporting information).

We showed that the SLS brain lipidome is profoundly altered as fatty alcohols cause accumulation of almost all ether lipid classes in both white and gray matter with a concomitant reduction in non‐ether lipids. The accumulating fatty alcohols most likely drive the enhanced synthesis of ether lipids and etherphospholipids with a characteristic pattern that is likely dictated by the tissue‐dependent aldehydes that are normally processed by FALDH (Figure 6). Ether lipids, including plasmalogens, are ubiquitously found throughout human tissues and are known to be especially important in brain, heart and spleen.^16^ For most accumulating ether(phospho)lipid classes, the concentration in SLS white matter was much higher than in SLS gray matter (Figure 4). The neutral ether lipid and ether phospholipid classes with the highest fold changes between SLS and control, both in white and gray matter, were DG[O], TG[O], PC[O], and LPC[O]. The TG[O] increase was especially remarkable as TG[O] species normally are present in low levels in the brain. The accumulating DG[O] is probably converted to TG[O] as the amount of CDP‐ethanolamine/CDP‐choline generated by the Kennedy pathway needed for the conversion of DG[O] to PE[O] and PC[O], respectively, is limiting. Alternatively, the altered fatty alcohol/fatty acid composition of DG[O] could be incompatible with the substrate specificities of downstream biosynthetic enzymes. TG[O] is the most proximal metabolite to channel accumulating DG[O], the primary product of ether lipid biosynthesis before headgroups are added. Remarkably, PE[O] and LPE[O] were not significantly increased as a group in the SLS brain. Brain contains high PE[O] content, especially white matter, and 50% to 100% of PE[O] are plasmalogens.^32^ The plasmalogen synthesis is regulated by post translational degradation of FAR1,^33^ which is the rate‐limiting enzyme for fatty alcohol production from acyl‐CoAs. As the supply of fatty alcohols is high in SLS, plasmalogen synthesis will proceed normally, which leads to degradation of FAR1 that in turn limits PE[O] production. This possibly explains the relatively normal levels of PE[O] in SLS brain. If, as we suggest above, fatty alcohols are formed from reduction of aldehydes originating from catabolism of 2‐hydroxy‐fatty acids, sphingosine‐1‐phosphate and plasmalogens, this formation is not FAR1‐regulated and represents an uncontrolled influx of fatty alcohols into ether lipid synthesis which could explain the expansion of the ether lipid pool in SLS (Figure 6).

The accumulation of TG[O] has also been seen in cultured SLS keratinocytes,^20^ raising the possibility that this unique lipid profile links the pathogenesis of brain and skin symptoms that are so characteristic of this disease. The critical functions of these two organs depend on the formation of multilamellar membranes in myelin and in the stratum corneum. In SLS skin, ultrastructural studies demonstrate that these stacked membranes are reduced in number and interrupted by lipid deposits, resulting in a leaky epidermal water barrier and the dry scaly appearance of ichthyosis.^34^ Myelin membranes may be similarly perturbed by accumulation of these same lipids.

In addition to the abnormal ether phospholipid metabolism, neutral lipid metabolism was also disturbed in the SLS brain. We found increased concentrations of TG and CE which, in addition to TG[O], may also contribute to the “lipid peaks” on brain MR spectra of SLS patients and the lipid droplets seen in the histological study of the SLS brain. Patients with other disorders that lead to TG accumulation, that is, defects in *DDHD2* and Chanarin‐Dorfman syndrome, have similar lipid resonances in brain MR spectra.^35^, ^36^

An abnormal low concentration of ether lipids is well known in some peroxisomal diseases with obvious neurometabolic consequences for the brain.^36^ In contrast, increased concentrations are found in SLS. Recently, Vaz et al described *PCYT2* mutations that also heavily impact ether (phospho)lipid biosynthesis.^37^ Patients developed a progressive para‐ or tetraparesis with intellectual disability. In line with the metabolic role of the PCYT2 enzyme (catalyzing the rate limiting step of CDP‐ethanolamine biosynthesis for both PE and PE[O] [see also Figure 6]), lipidomics in fibroblasts showed significant accumulation of neutral lipids (TGs), ether lipids (DG[O], TG[O]), and ether phospholipids (LPC[O], PC[O]). The fibroblast lipid profile in *PCYT2* deficiency is reminiscent of that found in SLS brain. The brain lipid profile in patients with *PCYT2* deficiency has not been analyzed but specific differences with SLS may be anticipated.

The levels of the regular phospholipids PC, PS, PI, PG, and PE and their lyso‐forms were, if at all, mildly affected in SLS brain. This is to be expected as fatty alcohols do not play a role in the biosynthesis of these phospholipids. Some of these phospholipids, like PS, LPE and PE even showed a tendency toward a decreased concentration in SLS which could be caused by the consumption of CDP‐ethanolamine/CDP‐choline to synthesize PE[O] and PC[O]. Another possible explanation might be fatty aldehyde toxicity, causing inactivity of specific enzymes responsible for the production of these lipids. This mechanism was described in a mouse study in SLS, where the enzymatic activity of fatty acid 2‐hydroxylase (FA2H activity) was affected by fatty aldehyde accumulation.^38^ We detected no increase in free fatty aldehydes in the SLS brain and very long chain aldehydes were not seen. The storage time of the brain samples before analysis may have played a role in this. It is possible that these highly reactive lipids have formed covalent adducts with amino‐containing molecules and thus escape detection^39^ or were metabolized to fatty alcohols.^40^ Lastly, we did not see any leukotrienes and phytol, lipids that have been implicated in SLS pathophysiology. Phytol is probably processed in the liver and may never reach the brain.^41^ Leukotrienes are biologically very active lipids and presumably have concentrations below the detection limit and are not detected in our lipidomics platform.

The main limitation of this study is the fact that only one SLS brain and one replica as control could be included. Obtaining a single SLS‐brain that also was suited to perform biochemical studies was a unique opportunity. Equally so it has been very hard to obtain suitable control brain tissue (with same sex, age, no brain disease, and the brain postmortem not processed in formalin or otherwise).

In conclusion, we have found a severely disturbed lipid profile in both white and gray matter of the SLS brain, affecting both neutral ether lipids and ether phospholipids along with changes in neutral lipid metabolism. Taken together our data suggest that the brain disorder in SLS results from the accumulation of many different lipid species, especially fatty aldehydes, fatty alcohols, triglycerides and ether (phospho)lipids. Our data show that, at the biochemical level, SLS is not only a white matter but also a gray matter disease. Additionally, the histopathological study suggests that astrocyte dysfunction might play a pivotal role in the underlying disease mechanism, especially contributing to an impairment of myelin maintenance.

## CONFLICT OF INTEREST

The authors declare no potential conflict of interest.

## ETHICS APPROVAL

The study was performed according to the tenets of the declaration of Helsinki (2013 revision), and was approved by the Regional Committee on Research Involving Human Subjects.

## INFORMED CONSENT

Prior written consent was given by the guardian of the patient after decease; and by the healthy control patient herself before decease.

5

## Supporting information


**Data S1.** Materials and methods

**Data S2.** Supporting Information

**Data S3.** Supporting Information

**Data S4.** Supporting Information

**Data S5.** Supporting Information

**Data S6.** Supporting Information

## Data Availability

Data of the study were uploaded as Supporting Information.
