# Supplementary material for: Disturbed brain ether lipid metabolism and histology in Sjögren‐Larsson syndrome
Source: J Inherit Metab Dis. 2020 Jul 9;43(6):1265–78. doi: 10.1002/jimd.12275 (PMC7689726; doi:10.1002/jimd.12275)
Supplement: Supplementary file 3 — Data S3. Supporting Information [file JIMD-43-1265-s003.docx]

**Materials and methods**

A case-control study was performed on postmortem brain tissues of a 65-year-old female patient with genetically confirmed SLS and a 65-year-old female control patient.

*Study subjects and tissue processing*

Half of the SLS brain was processed for histological studies at the Born Bunge Institute (University of Antwerp, Antwerp, Belgium) after 4% formalin fixation and paraffin embedding. The other half was cut into 1-centimeter-thick coronal slices, and stored at -80 °C for biochemical analysis. The control brain, obtained from the department of Anatomy of the Radboud University Medical Center one day after death, was sliced and stored in the same way.

For lipid analyses, small 10 mg wet weight samples of neighboring white and gray matter were cut out from the occipital coronal slices, from both the SLS and control brain. We have chosen this location for sampling because in vivo MR spectroscopy studies in SLS patients have previously demonstrated the highest ‘lipid peaks’ in these occipital white matter regions ^1, 2^.

*Histopathological analysis*

Formalin-fixed, paraffin-embedded tissue sections were cut (5 µm-thickness) and stained with Hematoxylin&Eosin (H&E) and immunostained ^3^ for proteolipid protein (PLP; AbDSerotec, 1:3000), glial fibrillary acidic protein (GFAP; Millipore, 1:1000), S100β (Sigma, 1:200) and CD68 (Dako, 1:3000). Positive controls and negative controls (by omitting the primary antibody) were included to verify the specificity of the immunohistochemical labelling. Images were acquired on a Leica Microsystems DM6000B microscope.

*Targeted analysis of fatty acids, fatty aldehydes and fatty alcohols*

Fatty alcohols and fatty aldehydes were measured using a targeted approach at the laboratory in the University of Nebraska Medical Center (Omaha, NE, USA). Brain tissues (5 mg) were homogenized in 900 µl water. For fatty acid analysis, lipids were extracted from homogenates and washed according to Folch ^4^. Total fatty acids were measured by GC-MS ^5^. For fatty aldehyde analysis, tissue homogenates (400 µl) were treated directly with pentafluorobenzyl hydroxylamine (PFBHA) and PFB-oxime derivatives of free fatty aldehydes were analyzed by GC-MS as described ^6^. Pentadecanal (10 µg) was used as internal standard. Fatty aldehydes were identified by their characteristic m/z 181 fragment-ion using authentic standards synthesized as described ^7^. For fatty alcohol analysis, tissues (5 mg) were homogenized in 300 µl water. After removal of 50 µl for protein determination, the remaining homogenate was extracted with 1 ml methanol for 1 hour. Pentadecanol (0.1 µg) was added as internal standard. Free fatty alcohols were extracted from the methanol/water twice using 1 ml hexane. Hexane extracts were combined, dried under nitrogen and treated with pentafluorobenzoylchloride (PFB-Cl). The PFB derivatives were analyzed by GC-MS as described ^8^. Fatty alcohols were normalized on protein.

*Untargeted lipidomics analysis*

Lipidomics analysis was performed as described by Herzog et al. ^9^ at the Core Metabolomics Facility (Amsterdam University Medical Center, the Netherlands). Brain samples were measured in triplicate using three pieces of the same brain samples that were processed independently. The lipid classes included bis(monoacylglycerol)phosphates (BMPs), ceramide-1-phosphates (C1Ps), cholesteryl esters (CEs), ceramides (Cer[d]s), cardiolipins (CLs), diacylglycerols (DGs), 1-alkyl-2-acylglycerols (DG[O]s), dilysocardiolipins (DLCLs), dihexosylceramides (Hex2Cers), hexosyl­ceramides (HexCers), (lyso)phosphatidic acids ((L)PAs), (lyso)phosphatidylcholines ((L)PCs), (lyso)phosphatidylcholine etherphospholipids ((L)PC[O]s), (lyso)phosphatidylethanolamines ((L)PEs), (lyso)phosphatidylethanolamine etherphospholipids ((L)PE[O]s), (lyso)phosphatidylglycerols ((L)PGs), monolysocardio­lipins (MLCLs), phosphatidylinositols (PIs), phosphatidylserines (PSs), sphingo­sines/sphin­gosine-1-phosphates (S1Ps), sphingomyelins (SMs), sulfatides (SM4s), triacylglycerols (TGs) and 1-alkyl-2,3-diacylglycerols (TG[O]s). The PE[O] and PC[O] phospholipid groups contained contri­bu­tions from plasmanyl species and plasmenyl species (=plasmalogens). The approach is semi-quantitative by the addition of an internal standard per lipid class of interest with a known quantity and assuming similar response in MS. Therefore the quantity of the lipids is expressed as relative abundance (nmol/mg protein). For the annotation of lipid species, the lipidmaps nomenclature ([www.lipidmaps.org](http://www.lipidmaps.org)) was used as much as possible.^10^ Individual molecular lipid species are annotated as ‘lipid subclass’ (total fatty acyl chain length: total number of unsaturated bonds); TG(50:4) thus means a triacylglycerol with a total of 50 carbon-atoms and 4 double bonds in the three fatty acid side-chains (Figure 1). Lipid species with an ether bond in the *sn-*1 position, are annotated as ‘lipid subclass’ [O]; PE[O] thus being an 1-*O-*alkyl/alkenylphosphatidyl­ethanolamine.

For the plasmanyl/plasmenyl distribution analysis, 75 µl of the final brain lipid extract of the different samples (white and gray matter controls / SLS patient, all n=1, 8 samples in total) was either combined with 75 µl of methanolic HCl (0.5 M, Sigma) or with 75 µl of methanol and incubated at room temperature for 30 minutes. Samples were taken to dryness under a nitrogen stream at 40°C and resuspended in chloroform/methanol (1:1, v/v). This sample was used for lipidomics and selected PE[O] and PC[O] species that could be detected in at least 7 of the 8 samples were included in the analysis. Bar graphs were made for intensities (see first tab of supplement 2 for legend) of these species which were evaluated for 1. plasmanyl (acid stable)/plasmenyl (acid labile) or a mixed pattern and 2. whether this pattern was different between control and SLS.

*Mass spectrometry imaging*

Tissue sections (10 µm thickness) from the SLS and control brains were cut, thaw-mounted on indium-tin-oxide (ITO) coated glass slides, and stored at -80°C until analysis. Prior to analysis, tissues were equilibrated to room temperature by freeze-drying, and homogeneously coated with 20 mg/mL 2,5-dihydroxybenzoic acid (DHB) dissolved in 50/0.1/49.9 (% v/v/v) acetonitrile/trifluoroacetic acid/deionized water using a SunCollect pneumatic spraying system (6 layers: 1: 10 µL/min, 2: 20 µL/min, 3: 30 µL/min, 4+: 40 µL/min) resulting in a matrix coverage of approximately 2.2 µg DHB/mm^2^.

Matrix-assisted laser desorption/ionization mass spectrometry imaging (MALDI-MSI) was performed on a 9.4 T SolariX xR mass spectrometer (Bruker Daltonics, Germany) in positive-ion mode, using a 500 Hz laser repetition rate, 500 shots per spot and 100x100 μm^2^ pixel size. Data was acquired in a *m/z* range from 100-1500 Da with a 1 million data point transient (0.36 s duration). Data acquisition was performed using ftmsControl and flexImaging 4.0 (Bruker Daltonics).

Data was loaded in SCiLS lab (v2016b, Bruker Daltonics), and regions of interest were created for white and gray matter areas. Peak picking on the root-mean squared normalized overall average spectrum was performed in mMass^11^ using a relative intensity threshold (rel.int ≥ 1.0 %). In SCiLS lab, a Receiver Operating Characteristic (ROC) analysis was performed (subset of 2500 spectra per group, area-under-curve (AUC) ≥ 0.85). Molecular features positive for the SLS brain were assigned with molecular identities by mass matching the measured *m/z* values with the LIPIDmaps database (https://www.lipidmaps.org; max. mass error 0.01 *m/z*, including H^+^, Na^+^ and K^+^ adducts).

*Statistics*

All biochemical analyses were measured in triplicate and values are given as means with standard deviations (SD). A Students t-test with post-hoc Bonferroni correction was used for statistical comparison between groups. A p-value <0.01 was considered statistically significant.

*Characteristics of patient and control*

The patient had a classical SLS phenotype with intellectual disability, spastic diplegia and generalized ichthyosis, and died aged 65 years. She also suffered from epilepsy with generalized seizures, and diabetes mellitus. The patient did not suffer from an extrapyramidal movement disorder and did not use zileuton. SLS was genetically confirmed by demonstrating compound heterozygous *ALDH3A2* mutations known to be pathogenic: c.943C>T and c.1297_1298delGA ^12^. The patient was previously described by Van Mieghem et al. in 1997 (case 1) ^13^, and had bilateral symmetrical white matter lesions on brain MR imaging. No MR spectroscopy was performed.

The age- and sex-matched control was a 65-year-old female who died of a myocardial infarction. She was known to have colon carcinoma, but there was no history of brain disease. She donated her body to science, and her brain was considered to be eligible as control brain for our study because of her sex, age and clinical absence of any neurological disorder.

*Ethics approval*

The study was performed according to the tenets of the declaration of Helsinki (2013 revision), and was approved by the Regional Committee on Research Involving Human Subjects.

**References**

1. van Domburg PH, Willemsen MA, Rotteveel JJ, et al (1999) Sjogren-Larsson syndrome: clinical and MRI/MRS findings in FALDH-deficient patients. *Neurology* 52: 1345-1352.

2. Willemsen MA, Van Der Graaf M, Van Der Knaap MS, et al (2004) MR imaging and proton MR spectroscopic studies in Sjogren-Larsson syndrome: characterization of the leukoencephalopathy. *AJNR Am J Neuroradiol* 25: 649-657.

3. Kevelam SH, Bugiani M, Salomons GS, et al (2013) Exome sequencing reveals mutated SLC19A3 in patients with an early-infantile, lethal encephalopathy. *Brain* 136: 1534-1543.

4. Folch J, Lees M, Sloane Stanley GH (1957) A simple method for the isolation and purification of total lipides from animal tissues. *J Biol Chem* 226: 497-509.

5. Rizzo WB, Watkins PA, Phillips MW, Cranin D, Campbell B, Avigan J (1986) Adrenoleukodystrophy: oleic acid lowers fibroblast saturated C22-26 fatty acids. *Neurology* 36: 357-361.

6. Kawai Y, Takeda S, Terao J (2007) Lipidomic analysis for lipid peroxidation-derived aldehydes using gas chromatography-mass spectrometry. *Chem Res Toxicol* 20: 99-107.

7. Valicenti AJ, Holman RT (1976) Oxidation of Long-Chain Alcohols to Aldehydes by Dipyridine Chromic Anhydride Complex. *Chem Phys Lipids* 17: 389-392.

8. Bowden JA, Ford DA (2011) An examination of pentafluorobenzoyl derivatization strategies for the analysis of fatty alcohols using gas chromatography/electron capture negative ion chemical ionization-mass spectrometry. *J Chromatogr B Analyt Technol Biomed Life Sci* 879: 1375-1383.

9. Herzog K, Pras-Raves ML, Vervaart MA, et al (2016) Lipidomic analysis of fibroblasts from Zellweger spectrum disorder patients identifies disease-specific phospholipid ratios. *Journal of lipid research* 57: 1447-1454.

10. Fahy E, Subramaniam S, Murphy RC, et al (2009) Update of the LIPID MAPS comprehensive classification system for lipids. *Journal of lipid research* 50 Suppl: S9-14.

11. Strohalm M, Hassman M, Kosata B, Kodicek M (2008) mMass data miner: an open source alternative for mass spectrometric data analysis. *Rapid Commun Mass Spectrom* 22: 905-908.

12. Willemsen MA, IJlst L, Steijlen PM, et al (2001) Clinical, biochemical and molecular genetic characteristics of 19 patients with the Sjogren-Larsson syndrome. *Brain* 124: 1426-1437.

13. Van Mieghem F, Van Goethem JW, Parizel PM, et al (1997) MR of the brain in Sjogren-Larsson syndrome. *AJNR Am J Neuroradiol* 18: 1561-1563.
