# Supplementary figures and images for: Disturbed brain ether lipid metabolism and histology in Sjögren‐Larsson syndrome
Source: J Inherit Metab Dis. 2020 Jul 9;43(6):1265–78. doi: 10.1002/jimd.12275 (PMC7689726; doi:10.1002/jimd.12275)

# Average mass spectra – CTRL tissues

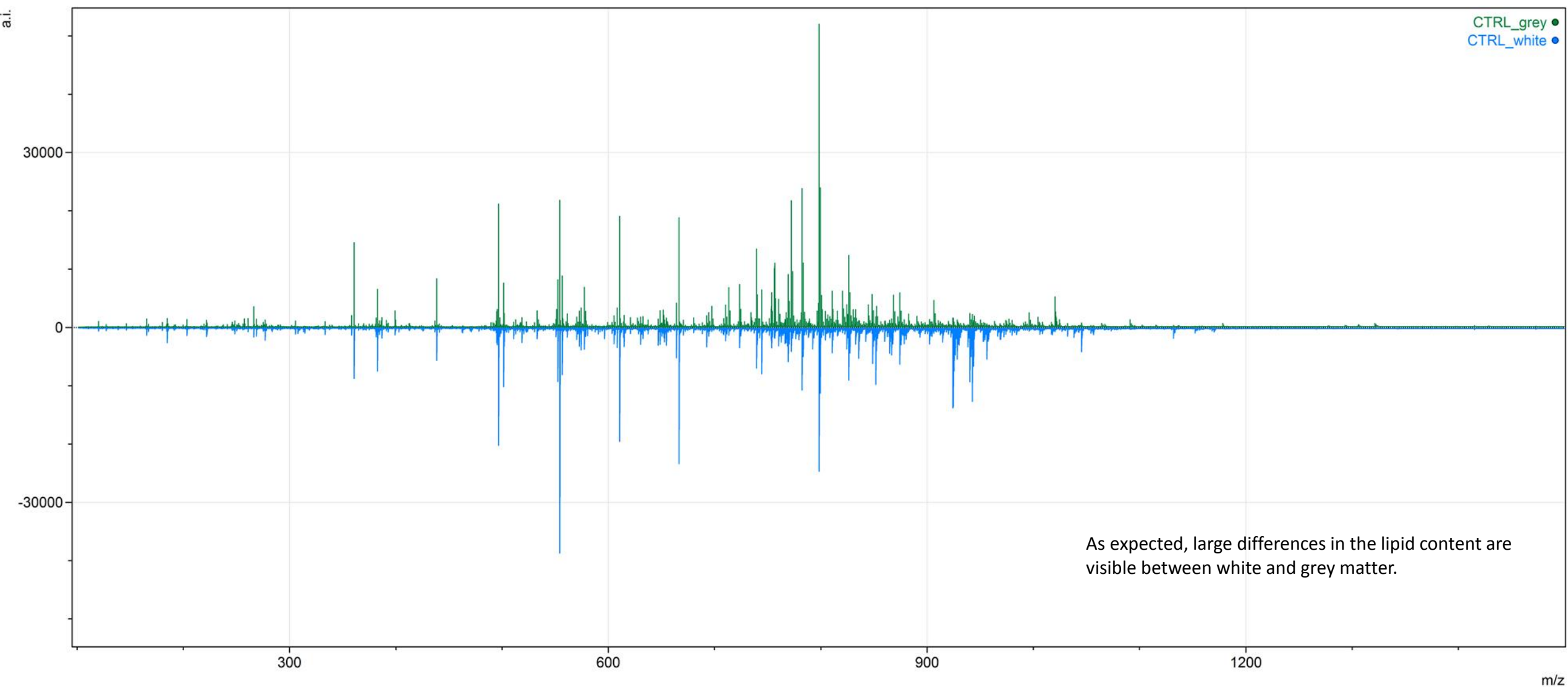

# Average mass spectra – SLS tissues

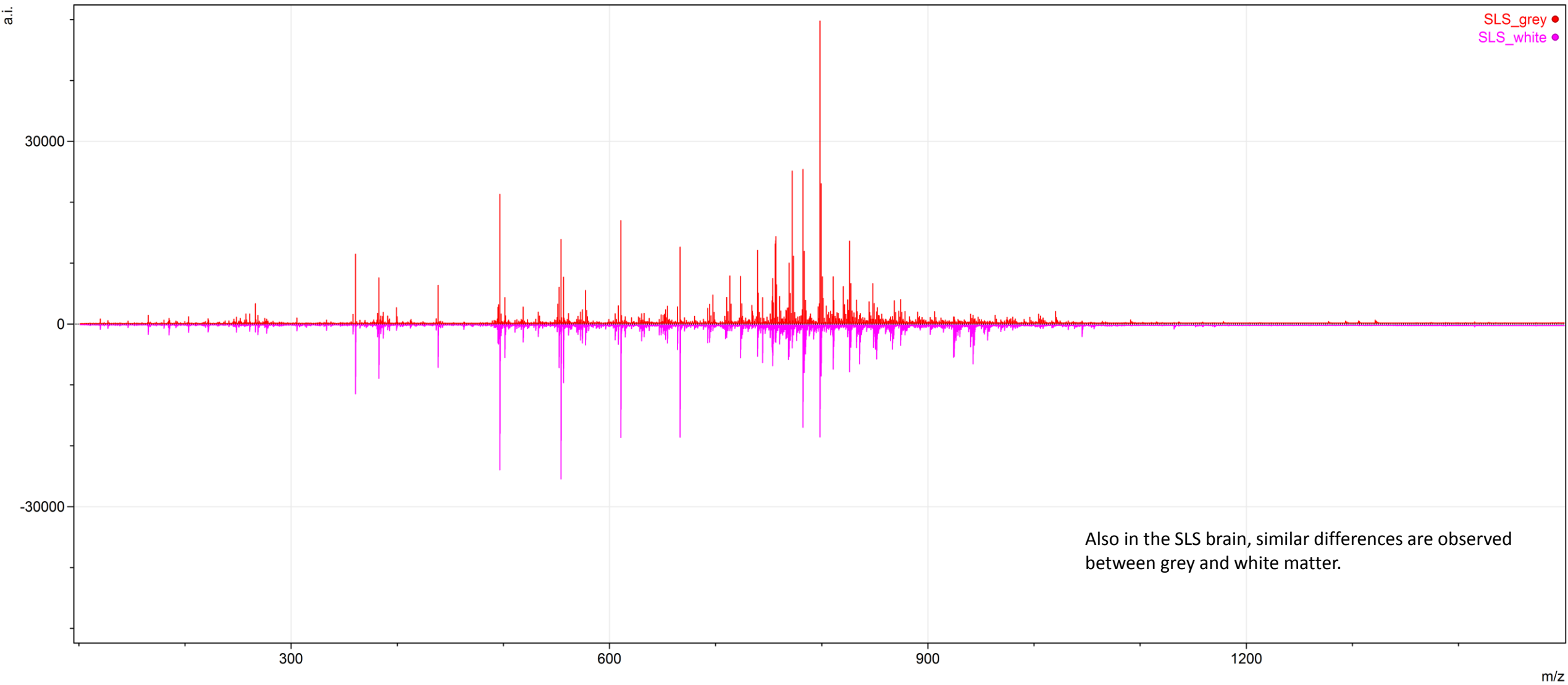

Supplement: Supplementary file 6 — Data S6. Supporting Information [file JIMD-43-1265-s006.pdf]
